# Differences in Fracture Healing Between Female and Male C57BL/6J Mice

**DOI:** 10.3389/fphys.2021.712494

**Published:** 2021-08-09

**Authors:** Melanie Haffner-Luntzer, Verena Fischer, Anita Ignatius

**Affiliations:** Institute of Orthopaedic Research and Biomechanics, University Medical Centre Ulm, Ulm, Germany

**Keywords:** fracture healing, bone healing, sex differences, estrogen, Wnt-signaling

## Abstract

**Background:**

Mice are increasingly used in fracture healing research because of the opportunity to use transgenic animals. While both, male and female mice are employed, there is no consensus in the literature whether fracture healing differs between both sexes. Therefore, the aim of the present study was to analyze diaphyseal fracture healing in female and male C57BL/6J mice, a commonly used mouse strain in bone research.

**Methods:**

For that purpose, 12-week-old Female (17–20 g) and Male mice (22–26 g) received a standardized femur midshaft osteotomy stabilized by an external fixator. Mice were euthanized 10 and 21 days after fracture and bone healing was analyzed by biomechanical testing, μCT, histology, immunohistochemistry and qPCR.

**Results:**

Ten days after fracture, Male mice displayed significantly more cartilage but less fibrous tissue in the fracture callus compared to Female mice, whereas the amount of bone did not differ. At day 21, Male mice showed a significantly larger fracture callus compared to Female mice. The relative amount of bone in the fracture callus did not significantly differ between both sexes, whereas its tissue mineral density was significantly higher in Male mice on day 21, indicating more mature bone and slightly more rapid fracture healing. These results were confirmed by a significantly greater absolute bending stiffness of the fractured femurs of Male mice on day 21. On the molecular level, Male mice displayed increased active β-catenin expression in the fracture callus, whereas estrogen receptor α (ERα) expression was lower.

**Conclusion:**

These results suggest that Male mice display more rapid fracture healing with more prominent cartilaginous callus formation. This might be due to the higher weight of Male mice, resulting in increased mechanical loading of the fracture. Furthermore, Male mice displayed significantly greater activation of osteoanabolic Wnt/β-catenin signaling, which might also contribute to more rapid bone regeneration.

## Introduction

Fracture healing is a highly regulated and complex process involving many cell types and signaling pathways, but remains insufficiently understood. To analyze the bone healing process in molecular detail, animal models in which it is possible to induce or delete specific factors involved during the healing cascade are needed. For this reason, mice have become increasingly popular for fracture healing research in recent years, because there are manifold transgenic strains available. Thereby, it has to be taken into consideration that bone regeneration differs between young and old mice (Lu et al., 2005; Lu et al., 2008; Haffner-Luntzer et al., 2016b; Clark et al., 2017; Josephson et al., 2019) and between different strains (Manigrasso and O’Connor, 2008; Haffner-Luntzer et al., 2016b). Regarding sex differences, the literature is strongly debated. Some authors report more rapid bone regeneration in male rats and mice compared to females (Mehta et al., 2011; Deng et al., 2020). However, there are also studies showing no sex-specific differences in murine fracture healing (Collier et al., 2020; Working et al., 2020). In many studies, data generated from female and male mice are pooled together, simply stipulating that there would be no sex-specific difference in the healing process.

However, it is known that sex hormones like estrogen have considerable effects on bone healing (Beil et al., 2010; Haffner-Luntzer et al., 2017; Fischer et al., 2018). Further, it has been shown that estrogen receptor signaling pathway is differentially regulated in male and female mice and that knockout of the estrogen receptor alpha in osteogenic cells showed different effects on bone mass in male and female mice (McCauley et al., 2003; Parikka et al., 2005). Likewise, males and females display different amounts of circulating Wnt/beta-catenin signaling inhibitors like Sclerostin and Midkine. Since the Wnt/beta-catenin signaling pathway is one of the most important osteoanabolic pathways, this might influence fracture healing as well as sex-hormone signaling.

There are also clinical indications that male patients display more rapid fracture healing and that women may have an increased risk for atrophic non-unions rather than hypertrophic non-unions as observed in males (Rupp et al., 2019; Li et al., 2020). By contrast, there are also clinical studies reporting no influence of sex on fracture healing in specific fracture types (Johnson et al., 2019; Lofrese et al., 2019; Morochovic et al., 2019). In general, body weight might be critically involved into bone regeneration processes, since local tissue strains are an important determinator of callus tissue development. It has been shown previously that if there are high stresses at the fracture area, mesenchymal cells are likely to form fibrous tissue, whereas osseous tissue is generated under low stress conditions. At intermediate stresses, mesenchymal cells will differentiate into chondrocytes and initiate cartilaginous callus formation, which initially bridges the fracture gap. Therefore, body weight differences between males and females might influence the healing process (Claes and Heigele, 1999; Claes et al., 2000, 2011).

The aim of this study was to analyze the similarities and differences in diaphyseal fracture healing between 12-week-old female and male C57BL/6J mice. We chose this mouse model, because this mouse model is frequently used in fracture healing studies.

## Materials and Methods

### Animals and Experimental Design

**FIGURE 1:**
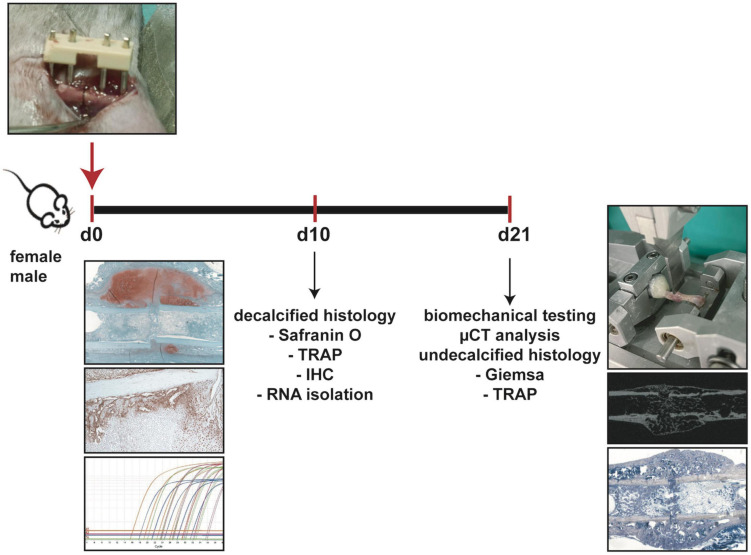
Experimental design. Female and male mice underwent femur osteotomy at 12 weeks of age. Mice were sacrificed either 10 days after surgery for decalcified histology (Safranin O staining, TRAP staining, immunohistochemical staining IHC and RNA isolation from sections) or 21 days after surgery for biomechanical testing, μCT analysis and undecalcified histology (Giemsa staining, TRAP staining).

All experiments were performed according to German Guidelines for Animal Research on the Protection of Animals as well as the ARRIVE guidelines and were approved by the local ethical committee (No. 1026, 1096, 1219, Regierungspräsidium Tübingen, Germany). Ten-week-old male and female C57BL/6J mice were purchased from Charles River laboratories. Following an acclimatization time period of 2 weeks, 12-week-old mice were subjected to a unilateral femoral midshaft osteotomy stabilized by an external fixator. Mice were euthanized at day 10 and 21 after fracture and bones were analyzed by biomechanical testing, μCT analysis, histology and gene expression analysis (Figure 1). Group size was 6–8 animals per group. Exact group size is specified in the figure legends.

Mice were maintained under standard laboratory conditions with up to five animals per cage on a 14 h light and 10 h dark rhythm with water and food available *ad libitum*. All mice received the same standard diet (R/M-H, V1535-300, Ssniff Spezialitäten GmbH). The female mice weighed 17–20 g on the day of the surgery, while the male mice weighed 22–26 g. Therefore, female mice were significantly lighter than male mice.

### Surgical Procedure

Surgery was performed under general anesthesia with 2% isoflurane (Forene, Abbott). The fracture healing model was previously described in detail (Rontgen et al., 2010). Briefly, a standardized osteotomy gap was created at the midshaft of the right femur using a 0.4 mm wire saw (RISystem) and stabilized by an external fixator (axial stiffness 3.0 N/mm, RISystem). The mice received as analgesia 25 mg/L tramalhydrochloride (Tramal, Gruenenthal GmbH) in the drinking water 1 day preoperatively until 3 days postoperatively. Post-operatively, the gap size was measured either in the histological sections (day 10) or by μCT analysis (day 21). Gap sizes ranged between 0.25 and 0.55 mm and did not differ between the groups.

### Biomechanical Testing

Fractured right and intact left femurs were harvested 21 days after surgery from the fractured mice, muscles were removed and specimens were stored in saline solution until biomechanical testing. 3-point bending testing was performed on the same day of euthanasia as described previously (Haffner-Luntzer et al., 2016a). Briefly, bones were embedded into aluminum cylinders at the proximal end and loaded in a materials testing machine (Zwick, Germany). The distal end rested freely on the testing machine (Figure 1). A maximum load of 4 N for intact and 2 N for fractured femurs was applied and the load-deflection curve was recorded. Bending stiffness was calculated from the slope of this curve. Relative bending stiffness was calculated using the values from the fractured and intact femur from the same animal. Some of the biomechanical parameters were already published in another context, because the mice used for this study served as control groups in previous studies (Heilmann et al., 2013; Bergdolt et al., 2017).

### μCT Analysis

Following biomechanical testing, the proximal end of the femurs was cut and specimens were fixed in 4% buffered formalin. Fixed samples were scanned using an isotrophic resolution of 8 μm and standard μCT settings of 200 mA and 50 kV in a μCT scanning device (Skyscan 1172). Fracture callus parameters were analyzed from the entire tissue between the two fractured cortices with a threshold of 642 mg hydroxyapatite/cm^3^. Bony bridging score was assessed according to clinical grading. The fractured femur was evaluated in two perpendicular planes. Each bridged cortex is counted as 1 point, therefore maximum reachable score is 4. A bony bridging score of 3 or 4 indicated successful healing.

### Histological Analysis

Following 48 h of fixation, bones from day 10 post fracture were decalcified and embedded in paraffin as described previously (Haffner-Luntzer et al., 2016a). Bones from day 21 post fracture were embedded undecalcified in methacrylate. Undecalcified histological sections allow better quantification of bone tissue in its native status, therefore we chose that type of embedding for the late time point where mostly bone is left in the fracture callus. Sections of 7 μm were cut from the middle of the fractured bone (area with largest callus size) and stained with Safranin O (decalcified) or Giemsa (undecalcified) or for tartrate-resistant alkaline phosphatase (TRAP). The amounts of bone, cartilage and fibrous tissue in the fracture callus were determined by Safranin O or Giemsa staining using image analysis software (Leica DMI6000 B; Software MMAF Version 1.4.0). Regions of interest were the fracture callus between the two inner pin holes on day 10 and between the two fractured cortices on day 21. A 500 × 500 μm box was counted within those regions. The number and surface of osteoblasts (NOb/BPm, ObS/BS, respectively) were determined in Safranin O- or Giemsa-stained sections. Osteoblasts are stained light blue in Safranin O staining and dark blue in Giemsa staining. They are defined as cubically shaped cells of intermediate size, residing on the bone surface. The number and surface of osteoclasts (NOc/BPm, OcS/BS, respectively) were determined after TRAP staining. Bone cells and surface were evaluated using Osteomeasure software (Osteometrics) according to ASBMR standards.

### Immunohistochemical Staining

Paraffin-embedded 7 μm longitudinal sections were prepared for immunohistochemical staining, using the following antibodies and dilutions: rabbit anti-mouse non-phospho (active) beta-catenin (#8814, CellSignaling 1:50), rabbit anti-mouse ERalpha (#sc-542, Santa Cruz 1:75), rabbit anti-mouse ERbeta (#sc-8974, Santa Cruz 1:40) and goat-anti rabbit IgG-biotin (sc-3840, Santa Cruz 1:100), and horseradish peroxidase (HRP)-conjugated streptavidin (Zytomed Systems). 3-Amino-9-ethylcarbazol (Zytomed Systems) was used as the chromogen. Sections were counterstained using haematoxylin (Waldeck). Species-specific non-targeting immunoglobulins were used as isotype controls.

### Gene Expression Analysis

RNA was isolated from 15 μm paraffin sections (three sections per mouse) using the RNEasy FFPE kit (Qiagen) according to the manufacturer’s instructions. The amount and purity of RNA was measured photometrically with a Tecan NanoPlate. Quantitative PCR was performed using the SensiFAST SYBR Hi-ROX One-Step Kit (Bioline) according to the manufacturer’s instructions. *B2M* was used as the housekeeping gene (F: 5′-ccc gcc tca cat tga aat cc-3′, R: 5′-tgc tta act ctg cag gcg tat-3′). Relative gene expression of *Axin2* (F: 5′-ATA AGC AGC CGT TCG CGA TG-3′ R: 5′-GCA ATC GGC TTG GTC TCT CT-3′), *Esr1* (F: 5′-tcc ggc aca tga gta aca aa-3′, R: 5′-cca gga gca ggt cat aga gg-3′) and *Esr2* (F: 5′-gag tag ccg gaa gct gac ac-3′, R: 5′-tct tca aaa tca ccc aga cc-3′) was calculated using the delta-delta CT method.

### Statistics

Data were tested for normal distribution using the Shapiro-Wilk normality test. Most datasets were normally distributed. Therefore, results are presented as bars with mean ± standard deviation. Statistical analysis was performed by Student’s *t*-test (GraphPad Prism 9). The level of significance was set at *p* < 0.05. Group size was 6–8, and was calculated based on the findings of a previous fracture healing study with the main outcome parameters of flexural rigidity and BV/TV in the fracture callus (Wehrle et al., 2015).

## Results

**FIGURE 2:**
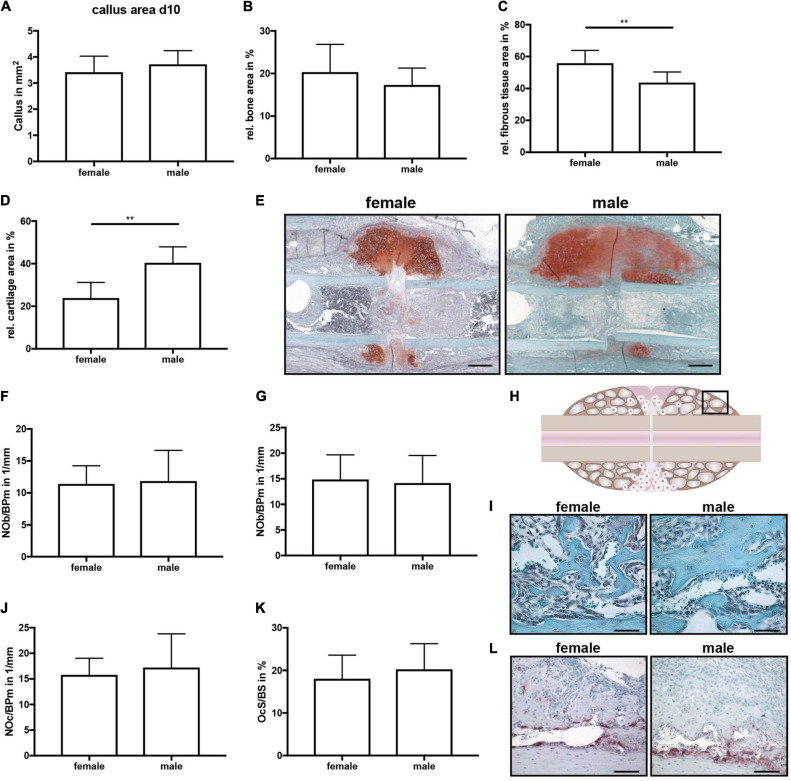
Histomorphometrical analysis of fractured femurs at day 10 after fracture. Analysis was performed of the entire fracture callus between the two inner pin holes. **(A)** Whole callus tissue area, **(B)** relative bone area, **(C)** relative fibrous tissue area and **(B)** relative cartilage area within the whole fracture callus at day 10 after fracture as determined by Safranin O staining. **(E)** Representative images from Safranin O staining of the fractured femurs. Scale bar=500μm. **(F)** Number of osteoblasts per bone perimeter and **(G)** osteoblast surface per bone surface in the bony fracture callus at day 10 after fracture as determined by Safranin O staining. **(H)** Schematic illustration of the region of interest (black box) for osteoclast and osteoblast analysis. **(I)** Representative images from Safranin O staining of the bony fracture callus. Scale bar = 50 μm. **(J)** Number of osteoclasts per bone perimeter and **(K)** osteoclast surface per bone surface in the bony fracture callus at day 10 after fracture as determined by TRAP staining. **(L)** Representative images from TRAP staining of the bony fracture callus. Scale bar = 50 μm. *n* = 8 (females) and *n* = 7 (males). Results marked with ** represent 0.01 > p < 0.001.

**FIGURE 3:**
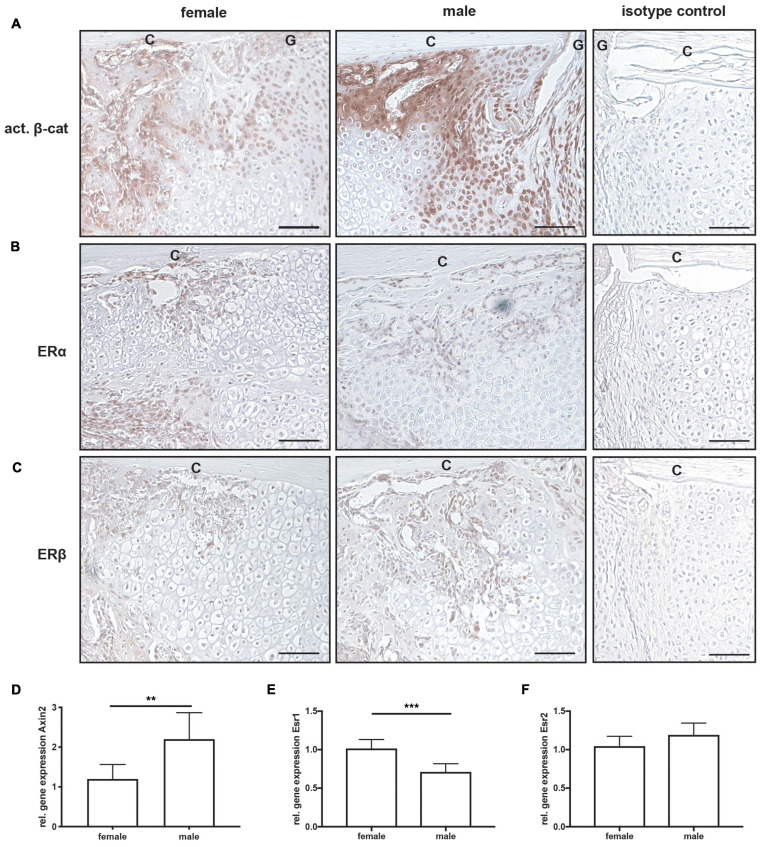
Immunohistochemical staining and gene expression analysis of fractured femurs at day 10 after fracture. Representative images from fractured femurs stained for **(A)** active β-catenin, **(B)** estrogen receptor α (ERα) and **(C)** estrogen receptor β (ERβ). Isotype control staining are shown as negative controls. Relative gene expression of **(D)** *Axin2*, **(E)** *Esr1* (ERα) and **(F)** *Esr2* (ERβ) as analyzed by qPCR from whole fractured femur sections. Scale bar = 150 μm. C, cortex; G, fracture gap. *n* = 7 per group. Results marked with ** represent 0.01 > p < 0.001 and *** 0.001 > p < 0.0001.

All mice displayed normal limb loading within the first 3 days after fracture. No animals were lost due to anesthesia or surgical issues. At day 10 after fracture, the callus size did not significantly differ between female and male mice (Figure 2A). Additionally, the bone content of the fracture callus did not differ between both sexes (Figure 2B). However, the fibrous tissue fraction was significantly lower, whereas the cartilage fraction was significantly higher in male mice (Figures 2C–E). There were no significant differences in osteoblast or osteoclast numbers or surface between female and male mice (Figures 2F–L). Immunohistochemical staining revealed that active β-catenin, a marker for activated Wnt-signaling, was highly expressed in osteoblasts and proliferating chondrocytes in the fracture callus, whereas hypertrophic chondrocytes displayed no or only less expression (Figure 3A). Although this expression pattern was found in both sexes, the staining intensity was much stronger in male animals. Furthermore, also the relative gene expression of the Wnt/beta-catenin signaling pathway target gene Axin2 was significantly increased in male mice (Figure 3D). ERα was expressed in osteoblasts and chondrocytes in the fracture callus of female and male mice, however, its expression particularly in hypertrophic chondrocytes was lower in male mice (Figure 3B). ERβ was expressed in osteoblasts and chondrocytes in the fracture callus with no obvious differences between both sexes (Figure 3C). Gene expression analysis of fractured femurs confirmed significantly lower ERα expression in male mice (Figure 3E). No differences were detected in ERβ gene expression (Figure 3F). Isotype control staining demonstrated specificity of the immunohistochemical staining (Figures 3A–C).

**FIGURE 4:**
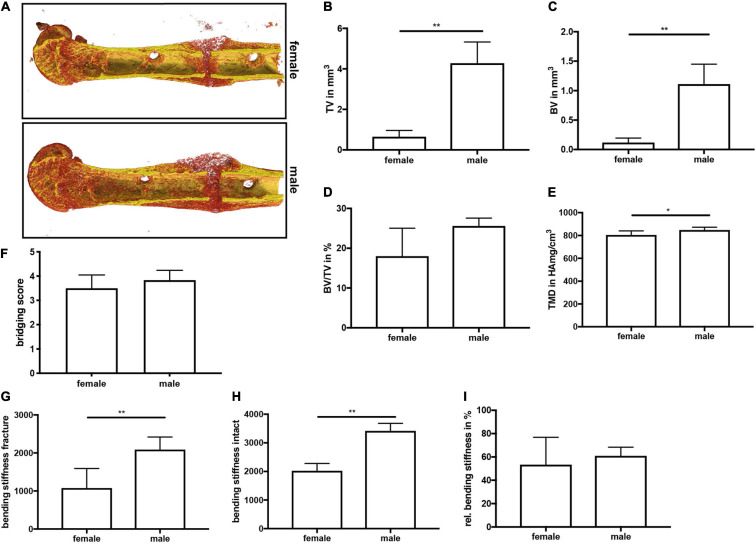
μCT analysis and biomechanical testing of fractured femurs at day 21 after fracture. **(A)** Representative 3D images of fractured femurs were generated automatically by the program CTVox and the standard transfer function for bone and metal. BMD calibration showing less mineralized areas in red, whereas highly mineralized areas are displayed in yellow. **(B)** Callus tissue volume, **(C)** bone tissue volume, **(D)** bone volume ratio and **(E)** tissue mineral density in the fracture gap at day 21 after fracture. **(F)** Bony bridging score. **(G)** Bending stiffness of fractured femurs, **(H)** bending stiffness of intact femurs and **(I)** relative bending stiffness of the fractured femur at day 21 after fracture. *n* = 6 per group. Results marked with ** represent 0.01 > p < 0.001.

**FIGURE 5:**
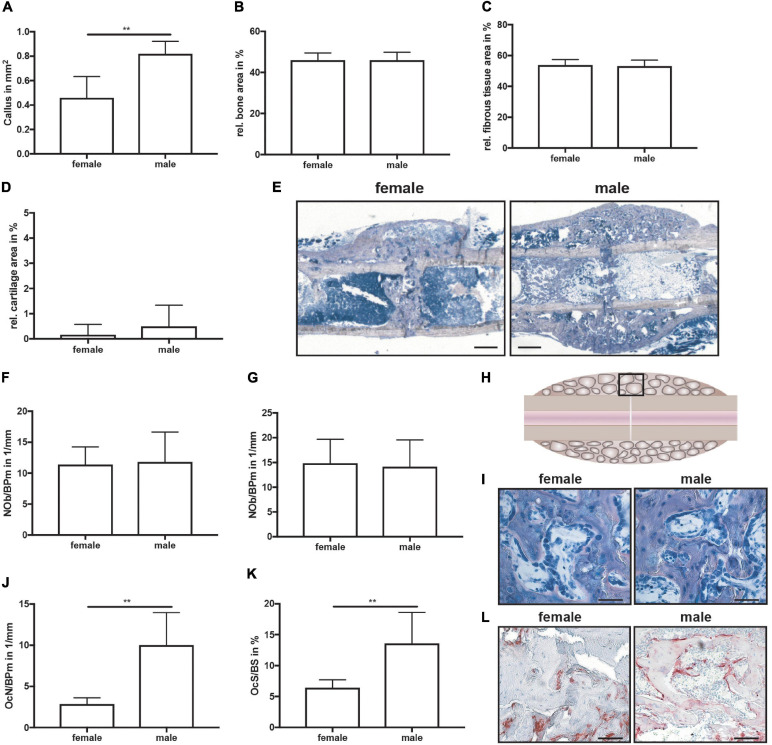
Histomorphometrical analysis of fractured femurs at day 21 after fracture. Analysis was performed of the entire fracture callus between the two fractured cortices (gap). **(A)** Whole callus tissue area, **(B)** relative bone area, **(C)** relative fibrous tissue area and **(D)** relative cartilage area in the whole fracture callus at day 21 after fracture as determined by Giemsa staining. **(E)** Representative images from Giemsa staining of the fracture callus. Scale bar = 500 μm. **(F)** Number of osteoblasts per bone perimeter and **(G)** osteoblast surface per bone surface in the bony fracture callus at day 21 after fracture as determined by Giemsa staining. **(H)** Schematic illustration of the region of interest (black box) for osteoclast and osteoblast analysis. **(I)** Representative images from Giemsa staining of the bony fracture callus. Scale bar = 50 μm. **(J)** Number of osteoclasts per bone perimeter and **(K)** osteoclast surface per bone surface in the bony fracture callus at day 10 after fracture as determined by TRAP staining. **(L)** Representative images from TRAP staining of the bony fracture callus. Scale bar = 50 μm. *n* = 6 per group. Results marked with ** represent 0.01 > p < 0.001.

On day 21, μCT analysis revealed a significantly larger fracture callus in male compared to female mice (Figures 4A–C). By contrast, the bone volume to tissue volume ratio did not differ between both sexes, whereas the tissue mineral density of the newly formed bone was significantly higher in male mice (Figures 4D,E). Furthermore, a bony bridging score of 3 or 4 indicated successful fracture healing in all animals with no significant differences between the groups (Figure 4F). Biomechanical testing analysis revealed a greater bending stiffness of fractured and intact femurs in male mice (Figures 4G–I). Bending stiffness was calculated from the slope of the load/deflection curve during 3-point-bedning. Relative bending stiffness was calculated using the values from the fractured and intact femur from the same animal. Histomorphometrical analysis confirmed that the callus was significantly larger in male mice (Figure 5A), whereas the relative bone area, fibrous tissue area and cartilage area did not differ (Figures 5B–E). At the bone trabeculae, the osteoclast number and surface were significantly greater in male mice compared with females (Figures 5J–L), whereas osteoblast number and surface did not differ between both sexes (Figures 5F,G).

## Discussion

The aim of the present study was to shed light on the question of whether the fracture healing process differs between female and male C57BL/6J mice. Our data suggest a slightly more rapid fracture healing with a more prominent cartilaginous callus formation in male compared to female mice.

On day 21, the time point of bony bridging of the fracture gap, the absolute bending stiffness of the fracture callus was significantly greater in male mice. However, because the absolute bending stiffness of the intact femur was also greater in male mice, relative values did not differ between both sexes. Male mice display stronger bones than female mice because of greater cortical width (Sims et al., 2002). The greater absolute bending stiffness of the fractured femurs of male mice might be caused by the significantly greater fracture callus size, which might result from the larger dimensions of the femur and greater mechanical loading of the fractured femur. Callus development highly depends on the interfragmentary strains in the fracture area (Claes and Heigele, 1999; Claes et al., 2000, 2011). The higher body weight in male mice might have resulted in a stronger deformation of the used semi-rigid external fixator during limb loading, resulting in greater interfragmentary strains. Larger fracture calli in male mice compared to female mice were also found in a study using a tibia fracture model with intramedullary stabilization (Deng et al., 2020), whereas others did not detect differences between male and female mice regarding callus size in a similar model (Collier et al., 2020).

Furthermore, we found that the relative bone volume in the fracture callus did not differ between female and male mice. However, male mice displayed higher tissue mineral density, indicating a more mature bone in the fracture callus and further supporting a slightly more rapid fracture healing process, although osteoblast number and activity did not differ. By contrast, osteoclast number and activity were significantly increased in the fracture callus of male mice. This is surprising, because one would suggest that more osteoclasts would lead to more rapid callus remodeling, whereas, male mice displayed larger fracture calli at this time point. It would be of interest to include more time points during the late phase of fracture healing to investigate the kinetics of osteoclast differentiation and callus remodeling. Deng et al. found more osteoclasts in the fracture callus of female mice 6 weeks after fracture (Deng et al., 2020). Therefore, it is likely that osteoclast formation and callus remodeling differ between both sexes.

During the intermediate phase of endochondral fracture healing at day 10 after surgery, male mice displayed a similar callus size compared to female mice, however, the cartilage content was significantly greater in male mice, whereas the amount of fibrous tissue was significantly lower. This indicates more rapid cartilaginous callus development in male mice. One reason might be the already mentioned differences in the biomechanical environment at the fracture site. Furthermore, on the molecular level, male mice displayed greater activation of the osteoanabolic Wnt/β-catenin signaling pathway in the fracture callus. Because inhibition of this pathway specifically in chondrocytes led to delayed cartilaginous callus formation during fracture healing (Huang et al., 2012), altered Wnt/β-catenin signaling might play a role here. However, a closer look into the expression of Wnt/β-catenin target genes would be necessary to determine possible molecular mechanisms. Another difference between male and female mice was found regarding ERα expression in the fracture callus. Male mice displayed significantly less ERα expression than female mice, particularly in hypertrophic chondrocytes. This might also account for the differences found in cartilaginous callus formation, because estrogen-dependent ERα signaling was shown to be important for the regulation of chondrocyte differentiation during endochondral ossification (Borjesson et al., 2013). Studies investigating mechanistic differences in fracture healing between male and female mice are generally lacking. One study suggested that male rats exhibit more mesenchymal stem cells in the bone marrow compared to female rats and that this might account for more rapid fracture healing in male rodents (Strube et al., 2009).

Clinical studies analyzing differences in fracture healing between male and female fracture patients are rather rare and results are difficult to interpret, because other influencing factors, including fracture type, degree of tissue trauma, body weight and clinical manifestation of osteoporosis, frequently differ between men and women. It was shown for some types of fractures that male patients displayed more rapid fracture healing and women had an increased risk for atrophic non-unions rather than hypertrophic non-unions in males (Rupp et al., 2019; Li et al., 2020). One clinical study reported significantly increased blood levels of the Wnt/β-catenin-signaling inhibitor Sclerostin in male geriatric hip fracture patients compared to female age-matched patients, whereas the concentrations of another Wnt/β-catenin-signaling inhibitor, Dickkopf-1 were reduced (Dovjak et al., 2014). When comparing male fracture patients to postmenopausal female fracture patients, men displayed significantly lower systemic levels of the inflammatory cytokine and Wnt/β-catenin-signaling inhibitor Midkine during long bone fracture healing (Fischer et al., 2018). Concluding, systemic concentrations of Wnt/β-catenin-signaling inhibitors appear to be different between male and female fracture patients, however, the underlying mechanisms have to date been minimally investigated.

In summary, the present study demonstrated specific differences in the endochondral fracture healing process between male and female mice, with male mice displaying a more rapid fracture healing process. Limitations of this study are that we did not analyze further time points during fracture healing and that we focused on only specific pathways which we hypothesized might be altered between the two sexes, namely Wnt/β-catenin and ER signaling. Further unbiased analyses of other pathways involved in the fracture healing process would be desirable. Nevertheless, this study suggests that the sex of the mice is relevant in fracture healing research and that data from male and female mice should not be pooled together.

## Data Availability Statement

The original contributions presented in the study are included in the article/supplementary material, further inquiries can be directed to the corresponding author/s.

## Ethics Statement

The animal study was reviewed and approved by Nos. 1026, 1096, 1219, Regierungspräsidium Tübingen, Germany).

## Author Contributions

MH-L and VF conducting experiments. MH-L and AI: substantial contributions to research design. MH-L, VF, and AI interpretation of data. MH-L: drafting the manuscript. All authors revising the manuscript critically and approval of the submitted version.

## Conflict of Interest

The authors declare that the research was conducted in the absence of any commercial or financial relationships that could be construed as a potential conflict of interest.

## Publisher’s Note

All claims expressed in this article are solely those of the authors and do not necessarily represent those of their affiliated organizations, or those of the publisher, the editors and the reviewers. Any product that may be evaluated in this article, or claim that may be made by its manufacturer, is not guaranteed or endorsed by the publisher.
